# High-pressure Methanogenesis Reveals Metabolic Adaptation to Dissolved CO_2_ Limitation

**DOI:** 10.1264/jsme2.ME25066

**Published:** 2025-12-12

**Authors:** Taiki Katayama, Hideyoshi Yoshioka, Masaru K. Nobu

**Affiliations:** 1 Institute for Geo-Resources and Environment, Geological Survey of Japan, National Institute of Advanced Industrial Science and Technology (AIST), Tsukuba, 305–8567, Japan; 2 Institute for Extra-cutting-edge Science and Technology Avant-garde Research (X-star), Japan Agency for Marine-Earth Science and Technology (JAMSTEC), Yokosuka, 237–0061, Japan

**Keywords:** CO_2_ reduction, deep biosphere, high-pressure, methanogenesis

## Abstract

This study investigated the effects of elevated hydrostatic pressure on methane production and gene expression in a hydrogenotrophic methanogen isolated from subseafloor sediments at biogenic gas hydrate sites, with a focus on the implications of CO_2_ availability. Using high-pressure cultivation, the methane production rate decreased by 15% at 25 MPa, while a transcriptomic anal­ysis revealed the marked up-regulation of methyl-coenzyme M reductase and ATP synthase. These results suggest that methanogens compensate for pressure-driven constraints on CO_2_ utilization by increasing the expression of key methanogenic enzymes, underscoring the overlooked role of CO_2_ in deep biosphere microbial processes.

Deep marine sediments harbor a large fraction of the Earth’s microbial biomass (Magnabosco *et al.*, 2018) and constitute the largest methane reservoir on Earth (Milkov, 2004). Geochemical evidence indicates that methane stored as gas hydrates beneath deep ocean basins is mainly generated by methanogenic archaea (Kvenvolden, 1995; Milkov, 2005). Therefore, identifying the environmental factors controlling methanogenesis in subseafloor settings is fundamental to understanding global biogeochemical cycles.

A distinctive feature of deep marine sediments is elevated hydrostatic pressure, which enhances the solubility of the gaseous methanogenic substrates, H_2_ and CO_2_. Regarding H_2_, higher solubility under elevated pressure has been shown to promote the growth of hyperthermophilic methanogens by improving access to the substrate (Bernhardt *et al.*, 1988b; Miller *et al.*, 1988; Takai *et al.*, 2008). In contrast, greater CO_2_ solubility decreases culture medium pH, thereby suppressing methanogenic activity (Bernhardt *et al.*, 1988a). Dissolved CO_2_ is also hydrated and ionized to HCO_3_^–^, a process that is promoted under elevated pressure (Abe and Horikoshi, 1998). These pressure-driven changes in CO_2_ speciation may reduce its availability for methanogenesis in deep subseafloor sediments. However, this effect has yet to be investigated, partly because of the opposing impacts of H_2_ and CO_2_ solubilities.

To assess the effects of CO_2_ availability under elevated pressure on methanogenic activity, we used formate as an alternative substrate to H_2_/CO_2_ and employed Good’s buffer (HEPES) instead of bicarbonate buffering systems in the culture medium. Periodic sampling from a high-pressure cultivation system (Fig. 1) enabled measurements of formate concentrations, from which methane production was calculated stoichiometrically. Experiments focused on *Methanocalculus* sp. strain 1H1Hc7 (JCM 39199), the predominant hydrogenotrophic methanogen isolated from sediments in the biogenic gas hydrate area of the eastern Nakai Trough (Katayama *et al.*, 2022).

Strain 1H1Hc7 exhibits optimal methane production at 45°C (Katayama *et al.*, 2022), corresponding to *in situ* conditions at ~2,500‍ ‍m below sea level, given a sediment geothermal gradient of 0.03°C m^–1^ (Kanno *et al.*, 2014). To simulate these conditions, we cultured the strain under 0.1‍ ‍MPa (atmospheric), 13 MPa, and 25 MPa (Supplementary Methods). The formate consumption rate at 25 MPa was approximately 15% lower than that at atmospheric pressure, whereas no decrease was observed at 13 MPa (Fig. 2). Despite this difference, the final cumulative methane stoichiometrically produced reached nearly the same level (~5.7‍ ‍mM) under all conditions (Fig. 2). Cell densities were also similar between 0.1 MPa (8.4×10^7^±3.5×10^7^ [mean±s.d.] gene copy numbers mL^–1^) and 25 MPa (6.8×10^7^±4.0×10^7^ mL^–1^) after cultivation. These results indicate that a high pressure primarily imposed a kinetic limitation rather than a yield limitation on methanogenesis.

The transcriptome anal­ysis compared cultures at 0.1 and 25 MPa, using a two-fold change in expression and a *P*-value of 0.05 as cut-offs. Of the 2,327 genes encoded in the 1H1Hc7 genome, 79 were significantly up-regulated and 100 were down-regulated at 25 MPa (Supplementary Table S1). Under high-pressure conditions, genes involved in methanogenesis were markedly up-regulated, particularly those encoding methyl-coenzyme M reductase (MCR, 1.7–4.0 fold) and ATP synthase (ATPase, 1.8–22 fold) (Table 1). In contrast, genes associated with transcription and protein synthesis were down-regulated at 25 MPa (Supplementary Table S2).

These results suggest that strain 1H1Hc7 responded to the change in the chemical equilibrium of CO_2_ under high pressure. During methanogenesis, formate is initially oxidized to CO_2_, and this is followed by its reduction to CH_4_ (Supplementary Fig. S1). However, under elevated pressure, the CO_2_ produced may be rapidly converted into HCO_3_^–^ (Abe and Horikoshi, 1998), thereby reducing the pool of CO_2_ directly available for methanogenesis (Vorholt and Thauer, 1997). Based on calculations using the equation by Millero (Millero, 1995), the CO_2_ concentration available for methanogenesis is 29.96‍ ‍mM under atmospheric pressure, but only 0.71‍ ‍mM under 25 MPa, representing more than a 40-fold decrease. Under bicarbonate-free, HEPES-buffered conditions, formate provides the only carbon input for methane formation, and the stoichiometric mass balance (4HCOOH → CH_4_+3CO_2_+2H_2_O) holds throughout the incubation, reflecting a nearly identical amount of final cumulative methane between 0.1 and 25 MPa. Under these CO_2_-limited conditions, the up-regulation of the MCR complex may play a key role in maintaining the balance of‍ ‍methyl-group intermediates within the terminal steps of‍ ‍methanogenesis. The reaction catalyzed by methyl-H_4_MPT:HS-CoM methyltransferase (Mtr), which transfers the methyl group from CH_3_–H_4_MPT to HS–CoM, is coupled to Na^+^ translocation across the membrane and, thus, constitutes an energy-conserving step of the pathway (Gottschalk and Thauer, 2001) (Supplementary Fig. S1). When CO_2_ availability is restricted, the supply of CH_3_–H_4_MPT to the Mtr–MCR module decreases, potentially lowering both methyl flux and Na^+^-motive energy conservation. By increasing MCR abundance, cells may more effectively consume CH_3_–S–CoM, keeping its intracellular concentration low and thereby pulling Mtr-coupled methyl transfer forwards. This enhanced flux coupling between Mtr and MCR helps sustain the Na^+^ motive force and overall energy conservation under kinetically unfavorable, CO_2_-limited conditions. The concomitant up-regulation of ATPase under pressure further supports this adaptive mechanism by facilitating ion translocation and energy conservation associated with the downstream methyl-transfer reaction or may represent a broader cellular response to pressure stress beyond methanogenesis.

The expression profiles of other methanogenic en­zymes‍ ‍support this interpretation. Upstream C_1_-transfer enzymes—‍formate dehydrogenase (Fdh), formylmethanofuran dehydrogenase (Fwd), formylmethanofuran-tetrahydromethanopterin formyltransferase (Ftr), methenyl-H_4_MPT cyclohydrolase (Mch), methylene-H_4_MPT dehy­drogenase (Mtd), methylene-H_4_MPT reductase (Mer), and Mtr—were not significantly up-regulated (Supplementary Table S1). Since these reactions are near-equilibrium steps with small Gibbs free-energy changes (Thauer, 1998), increasing their enzyme abundance may not contribute to overcoming CO_2_-limited conditions. In contrast, MCR catalyzes the exergonic step of methanogenesis, allowing it to drive metabolic flux even under thermodynamically constrained conditions.

Carbonic anhydrase is a key enzyme in the CO_2_-concentrating mechanism, providing an efficient strategy for carbon acquisition in photosynthetic microorganisms under CO_2_-limited conditions (Aizawa and Miyachi, 1986). Although a gene encoding a putative carbonic anhydrase (1H1ca_01525) was identified in the 1H1Hc7 genome, its expression was similar between 0.1 and 25 MPa, suggesting that strain 1H1Hc7 did not rely on this mechanism under the conditions tested herein. In methanogens, carbonic anhydrase is proposed to catalyze cytoplasmic CO_2_ conversion to HCO_3_^–^ during acetoclastic methanogenesis (Alber and Ferry, 1994); however, its physiological function in hydrogenotrophic methanogens remains unclear.

Our *in vitro* observations have implications for deep subseafloor sediments where the majority of CO_2_ is converted to HCO_3_^–^. In these environments, formate, rather than H_2_, may serve as an important CO_2_ sink, favoring hydrogenotrophic methanogenesis. In support of this, four of the six strains of hydrogenotrophic methanogens, including strain 1H1Hc7, which were previously isolated from subseafloor sediments in the Nankai Trough (Katayama *et al.*, 2022), produced methane from formate as the sole substrate (Supplementary Table S3). In addition, 16S rRNA gene amplicon sequencing data indicate that formate-utilizing methanogens are more abundant than non-formate-utilizing methanogens (Supplementary Table S3). Consistently, formate-utilizing methanogenesis has been shown to dominate over H_2_-utilizing methanogenesis in hyperalkaline environments with extremely limited CO_2_ availability (Fones *et al.*, 2021). The present results underscore the previously underestimated role of CO_2_ availability in affecting methanogenic activity and gene expression in the deep biosphere.

## Citation

Katayama, T., Yoshioka, H., and Nobu, M. K. (2025) High-pressure Methanogenesis Reveals Metabolic Adaptation to Dissolved CO_2_ Limitation. *Microbes Environ ***40**: ME25066.

https://doi.org/10.1264/jsme2.ME25066

## Supplementary Material

Supplementary Material 1

Supplementary Material 2

## Figures and Tables

**Fig. 1.**
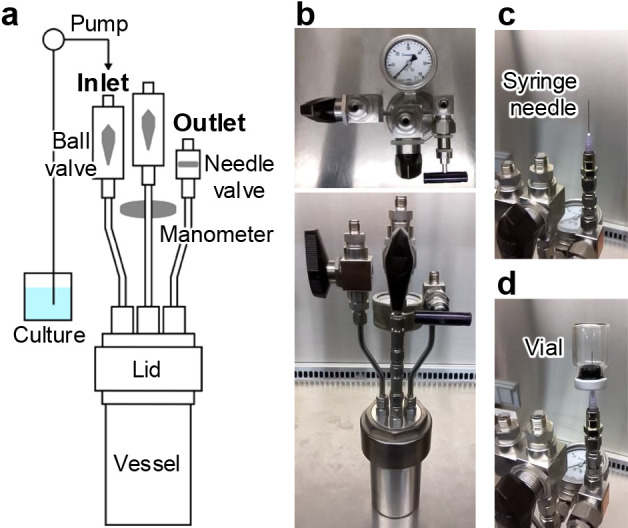
Schematic diagram (**a**) and photographs (**b, c, and d**) of the high-pressure cultivation system. A vial was set to a syringe needle connected with the outlet port to harvest the culture that was subsequently used for the quantification of formate concentrations (**c and d**).

**Fig. 2.**
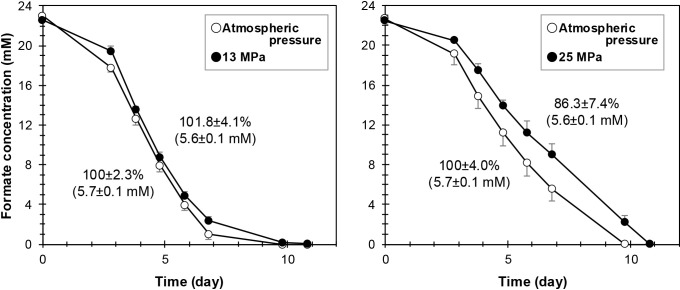
Effects of hydrostatic pressure on formate consumption and methane production in *Methanocalculus* sp. strain 1H1Hc7. Data are presented as the means and standard deviations (error bars) of quadruplicate experiments. The figure also shows methane production rates, with means and standard deviations, relative to those observed under atmospheric pressure conditions, and the final amounts of methane produced (mM, in parentheses) calculated based on the chemical formula representing the conversion of a quarter mole of methane from formate.

**Table 1.** List of genes significantly up-regulated under high pressure conditions. Expression levels of *Methanocalculus* sp. strain 1H1Hc7 genes in three replicates are shown as RPKM values, which were further normalized to the median expression level of all genes with mapped transcripts (N. RPKM).

Locus	Function	N. RPKM in 0.1 MPa			N. RPKM in 25 MPa		*P*-value
		A.V.	S.D.		A.V.	S.D.	
1H1ca_00514	Aquaporin AqpM	2.17	0.31		9.19	1.14	0.009
1H1ca_00394	ATP-dependent DNA helicase Rep	0.57	0.04		1.17	0.17	0.029
1H1ca_01282	Cell wall-active antibiotics response protein	0.65	0.12		1.33	0.13	0.008
1H1ca_01239	Coenzyme F390 synthetase	0.74	0.08		1.7	0.08	0.001
1H1ca_01924	D-inositol-3-phosphate glycosyltransferase	0.6	0.07		1.24	0.17	0.026
1H1ca_00994	Deoxyribodipyrimidine photo-lyase	0.31	0.02		0.63	0.07	0.017
1H1ca_02150	indole-3-glycerol phosphate synthase	2.98	0.21		6.12	0.33	0.001
1H1ca_01754	Methyl-coenzyme M reductase subunit alpha	26.27	2.7		63.83	13.59	0.042
1H1ca_01750	Methyl-coenzyme M reductase subunit beta	41.91	4.75		124.18	23.68	0.028
1H1ca_01753	Methyl-coenzyme M reductase subunit gamma	23.65	3.03		55.04	11.79	0.047
1H1ca_01752	Methyl-coenzyme M reductase operon protein C	23.81	0.34		74.87	15.93	0.031
1H1ca_01751	Methyl-coenzyme M reductase operon protein D	30.19	3.89		84.78	16	0.029
1H1ca_02149	N-(5′-phosphoribosyl)anthranilate isomerase	3.67	0.25		8.6	0.62	0.006
1H1ca_00992	Phosphate uptake regulator, PhoU	2.25	0.26		5.98	1.24	0.036
1H1ca_00326	Potassium-transporting ATPase ATP-binding subunit	0.64	0.04		1.41	0.2	0.023
1H1ca_01032	Putative aminoacrylate hydrolase RutD	0.66	0.17		1.46	0.29	0.026
1H1ca_02280	Sensor histidine kinase	1.21	0.06		3.04	0.71	0.047
1H1ca_01311	Small archaeal modifier protein 3	3.14	0.33		7.96	0.98	0.015
1H1ca_00896	tRNA(fMet)-specific endonuclease VapC	2.8	0.49		6.8	1.2	0.033
1H1ca_02147	Tryptophan synthase alpha chain	2.95	0.3		6.35	0.8	0.02
1H1ca_02148	Tryptophan synthase beta chain	3.3	0.37		8.14	0.61	0.001
1H1ca_01312	Tungsten-containing aldehyde ferredoxin oxidoreductase	5.25	0.81		11.2	1.19	0.006
1H1ca_00037	V-type ATP synthase alpha chain	0.41	0.03		1.01	0.21	0.04
1H1ca_02292	V-type ATP synthase alpha chain	0.79	0.13		6.58	1.87	0.033
1H1ca_02293	V-type ATP synthase beta chain	0.8	0.08		5.37	1.41	0.03
1H1ca_02290	V-type ATP synthase subunit C	1.27	0.31		13.64	3.22	0.022
1H1ca_02294	V-type ATP synthase subunit D	2.06	0.39		6.49	0.47	0.001
1H1ca_02291	V-type ATP synthase subunit F	0.66	0.14		6.29	1.46	0.022
1H1ca_02286	V-type ATP synthase subunit G	4.99	1.1		31.93	3.26	0.005
1H1ca_02287	V-type ATP synthase subunit I	2.46	0.61		12.26	1.69	0.011
1H1ca_02289	V-type ATP synthase subunit E	1.64	0.48		19.88	4.32	0.018
1H1ca_02288	V-type ATP synthase subunit K	1.57	0.34		19.28	4.44	0.02
