# Supplementary material for: High-pressure Methanogenesis Reveals Metabolic Adaptation to Dissolved CO2 Limitation
Source: Microbes Environ. 2025 Dec 12;40(4):ME25066. doi: 10.1264/jsme2.ME25066 (PMC12727201; doi:10.1264/jsme2.ME25066)
Supplement: Supplementary file 1 — Supplementary Material 1 [file 40_25066_s1.pdf]

Supplementary Information for

High-pressure methanogenesis reveals metabolic adaptation to dissolved CO2 limitation

Taiki Katayama, Hideyoshi Yoshioka and Masaru K. Nobu

Correspondence to: Taiki Katayama, Katayama.t@aist.go.jp

This file includes:

Supplementary Fig. S1. The deduced pathway for the conversion of formate to methane in *Methanocalculus* sp. 1H1Hc7 based on its genome analysis.

Supplementary Table S3. Formate utilization of methanogens previously isolated from the subseafloor sediment at the eastern Nankai Trough.

Supplementary Methods

References

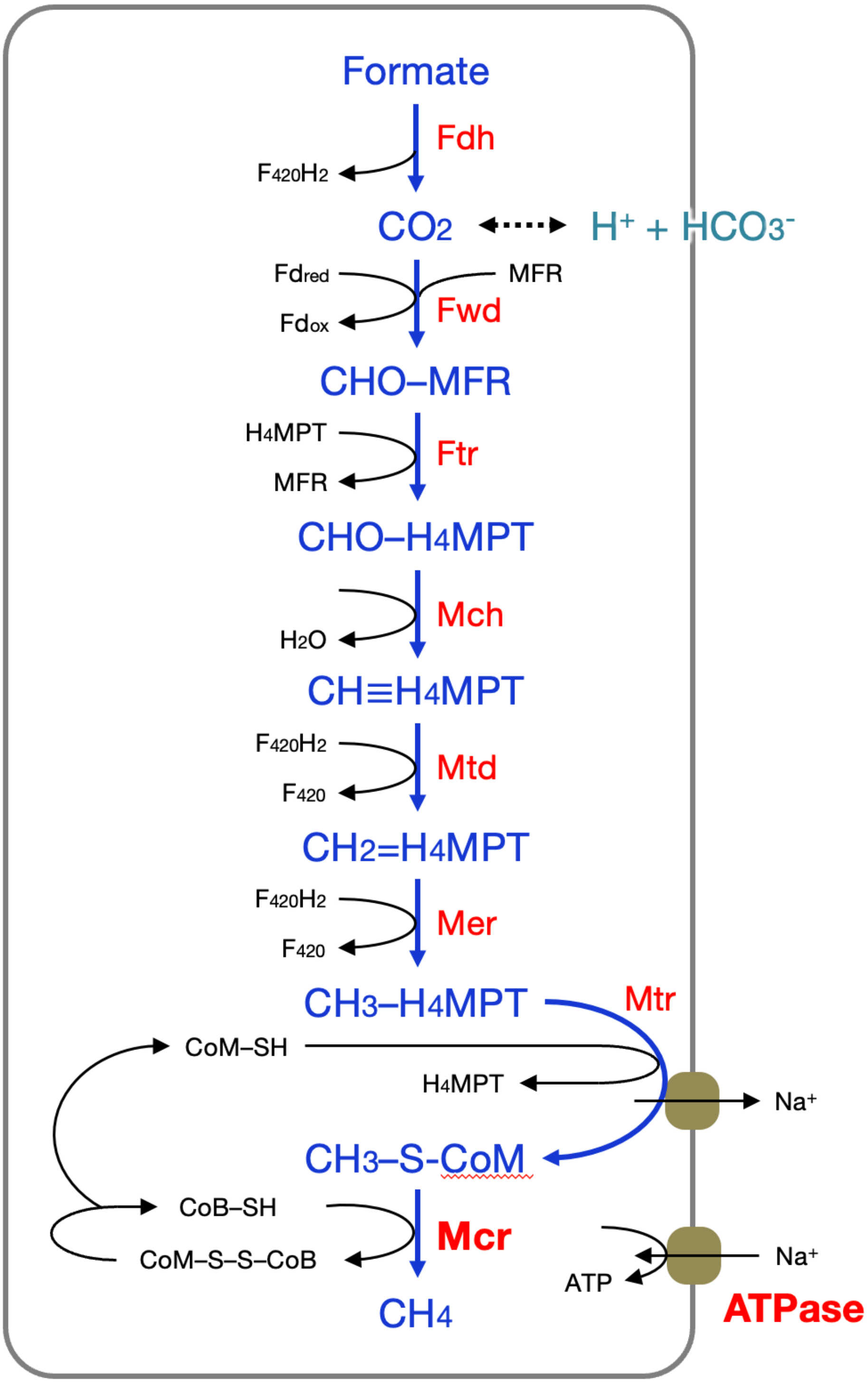

Supplementary Fig. S1. The deduced pathway for the conversion of formate to methane in *Methanocalculus* sp. 1H1Hc7 based on its genome analysis. The enzymes (red) that were highly expressed at elevated pressure are indicated by bold type. Broken arrow indicates the abiotic reaction, i.e., hydration and ionization of CO_2.

Supplementary Table S3. Formate utilization of methanogens previously isolated from the subseafloor sediment at the eastern Nankai Trough.

Strains	Closest species (% of sequence similarity)	Formate utilization	Abundance (%) relative to total methanogen sequences in the original sediment samples (mbsf)							
			1	11	43	259	282	295	300	307
1H1Hc7	Methanocalculus pumilus (99)	+	-	-	50	76	53	3.8	0.48	42
25XMc2	Methanoculleus marisnigri (99)	+	-	15	2.3	-	-	2.4	-	22
12X3c12	Methanoculleus taiwanensis (98)	+	-	-	-	-	-	-	-	-
1H2c2	Methanoculleus taiwanensis (95)	-	-	-	-	-	-	-	-	-
6TMc1	Methanoplanus limicola (100)	+	-	-	-	-	-	-	-	-
Mba6	Methanobacterium movens (99)	-	-	-	-	-	-	-	-	-

Supplementary Methods

Pre-culture conditions

Methanocalculus sp. strain 1H1Hc7 (JCM 39199) was cultivated in a sulfate-free saline mineral medium (pH 7.2) (Katayama and Kamagata, 2016), devoid of $NaHCO_3$ and supplemented with 40 mM sodium formate, 2 mM sodium acetate, 2 mM coenzyme M (2-mercaptoethanesulfonic acid), 0.2 mM titanium(III) citrate (as a reducing agent), and 40 mM HEPES. The pre-cultivation was performed at 25 °C under an N_2 atmosphere at atmospheric pressure. After five days of incubation, cells in the exponential growth phase were used as the inoculum for high-pressure cultivation experiments. In this pre-cultivation, growth was monitored by quantifying CH_4 in the headspace gas using gas chromatography equipped with a thermal conductivity detector (GC-8A; Shimadzu, Japan).

Cultivation at high hydrostatic pressure

High-pressure cultivation of *Methanocalculus* sp. strain 1H1Hc7 was conducted in a custom-built titanium pressure vessel (inner volume 500 mL; Fig. S1) equipped with inlet, outlet, and central ports fitted with ball and needle valves. The entire vessel and lid assembly were thoroughly purged with N_2 gas before sterilization. After the addition of 320 mL of basal saline mineral medium (previously N_2-flushed), the system was continuously flushed with N_2 for 20 min through the outlet port using a long needle to remove residual O_2, followed by an additional 3 min purge through the inlet and central ports. All ports were then closed, and the vessel autoclaved at 121 °C for 20 min.

All subsequent manipulations were carried out aseptically in a clean bench. After autoclaving, the outlet port was reopened, and N_2 was flushed through the system to maintain anaerobic conditions during addition of supplements. Sterile, N_2-flushed stock solutions of sodium formate (final 24 mM), acetate (2 mM), coenzyme M (2 mM), and titanium(III) citrate (0.2 mM) were sequentially injected via sterile syringes through the inlet port, followed by 20 mL of sterile, N_2-flushed basal medium to ensure homogeneity. Ten milliliters of exponential-phase pre-culture of strain 1H1Hc7 were then inoculated through the same port, and an additional 20 mL of medium was added to rinse the inlet line.

Prior to filling liquid within the vessel and pressurization, the entire pumping line of liquid pump (LC-20AD; Shimadzu Co. Ltd., Japan) was sterilized by circulating 75 % ethanol at 1 mL min^{-1} for 10 min, holding for 10 min, and flushing again for 5 min at 2 mL min^{-1}. The line was subsequently rinsed with sterile Milli-Q water (2 mL min^{-1}, 20 min) and filled with N_2-flushed, sterile medium of identical composition. The pump was connected to the vessel inlet, and medium of identical composition was delivered (2 mL min^{-1}) until all residual gas was expelled and the vessel was completely filled with liquid. Pressurization to 13 or 25 MPa was achieved by further pumping medium at 2 mL min^{-1} while monitoring the pressure gauge on the vessel and the pump.

After reaching the desired pressure, valves were closed and the vessel was detached from the pump and incubated statically at 25 °C. Sampling was performed periodically by connecting a sterile syringe and 5 mL vial to the outlet port to withdraw 0.5 mL of culture (Fig. 1c, d). The outlet valve was then closed, and the same volume of formate-free sterile medium (N_2-flushed) was introduced through the inlet to restore the internal pressure. Transient pressure drops during sampling were ≤ 2.5 MPa and returned to the setpoint within 1 min, as verified by the vessel manometer and pump pressure gauge. Control tests comparing serial sampling with endpoint-only incubation showed no significant differences in formate consumption of strain 1H1Hc7 (not shown), confirming that these transient pressure changes did not affect cell growth. For atmospheric-pressure cultures, sampling was performed by briefly pressurizing the vessel to 2.5 MPa with formate-free, N_2-flushed sterile medium. This temporary pressurization (within 1 min) enabled the culture liquid to be pushed out through the outlet port for sampling, as described above, while preventing air entry and maintaining anaerobic conditions.

After cultivation, sterility and purity were confirmed by microscopy (BX51; Olympus, Japan), which detected only autofluorescent methanogenic cells of strain 1H1Hc7, and bacterial 16S rRNA gene PCR amplification.

Formate concentration was measured using a Prominence HPLC system (Shimadzu) equipped with an electrical conductivity detector. Cumulative methane concentration was calculated based on

formate consumption using the stoichiometric equation: $4HCOOH \rightarrow CH_4 + 3CO_2 + 2H_2O$. For transcriptomic analysis, triplicate cultures were periodically supplemented with 20 mM formate under 0.1 and 25 MPa conditions at 10-day intervals, totaling 60 mM formate concentration. Cells were harvested in the middle phase of culture after the third formate addition, immediately post-depressurization, and subjected to DNA and RNA extraction as described below.

Genomic DNA extraction, sequencing, and analysis

Genomic DNA of 1H1Hc7 was extracted using the Allprep PowerFecal DNA/RNA kit (Qiagen, Netherlands) according to the manufacturer's instructions. DNA sequencing was performed using a GridION X5 system (Oxford Nanopore Technologies) and DNBSEQ G-400 system (MGI) at Bioengineering Lab. Co., Ltd. (Kanagawa, Japan).

Hybrid sequence assembly was accomplished using Unicycler v0.4.7 (Wick et al., 2017b; Wick et al., 2017a). Gene identification and annotations were performed using Prokka v1.14 (Seemann, 2014), followed by functional re-annotated using eggNOG-mapper v2.1.7 (Cantalapiedra et al., 2021) and CD-search v3.21 (Lu et al., 2020) with default settings.

The genome sequence of strain 1H1Hc7 has been deposited in GenBank under the accession number LC183832.

RNA extraction, sequencing and analysis

Total RNA was isolated using the Allprep PowerFecal DNA/RNA kit (Qiagen) with a bead-beating method, followed by purification using the RNeasy Mini kit (Qiagen) with DNase treatment (RNase-free DNase set, Qiagen) according to the manufacturer’s protocols.

RNA-seq was conducted at Bioengineering Lab. Co., Ltd. The MGIEasy RNA Directional Library Prep Set (MGI Tech Co., Ltd., Shenzhen, China) was used for library preparation after removing ribosomal RNA using the riboPool (siTOOLs Biotech, Munich, Germany) according to the manufacturer’s protocol. Following circularization using the MGIEasy Circularization Kit (MGI Tech

Co., Ltd.), DNA nanoballs were prepared using the DNBSEQ-G400 RS High Throughput Sequencing Kit (MGI Tech Co., Ltd.). RNA sequencing was performed on a DNBSEQ-G400 (MGI Tech Co., Ltd.), based on paired-end 100-bp sequencing.

Raw reads were quality-filtered and trimmed using fastp v0.23.4 (Chen et al., 2018), followed by mapping using bbmap v39.06 (https://sourceforge.net/projects/bbmap/) to 1H1Hc7 genome to calculate the relative transcript expression level, subsequently normalized as RPKM (Reads Per Kilobase of exon per Million mapped reads). The RPKM values were further normalized to the median expression level of all genes with mapped transcripts (Katayama et al., 2020), averaged (n=3), and compared between 0.1 and 25 MPa culture conditions.

The raw RNA-seq data have been deposited in the DDBJ Sequence Read Archive (DRA) under the accession number DRA018208.

Quantification of cell density

SYBR green-based real-time PCR was run on a CFX Connect real-time PCR detection system (Bio-Rad Laboratories Inc., USA) using the PowerUp SYBR green master mix (Applied Biosystems, USA) to quantify the cell density of 1H1Hc7. The primer paper, 1H_F (5'-AAGGAAACCCTGAGTGCCTGTC) and 1H_R (5'- AGACGCCTTTTGGTTGAGCCAAA) were designed from the 16S rRNA gene sequences of 1H1Hc7. The length of amplified products was 199 bp. Total DNA was extracted using an ISOSPIN Fecal DNA kit (NIPPON GENE, Japan). Standard curves for quantification were determined based on 10-fold serial dilutions of the target PCR products of 1H1Hc7 at known concentrations. All reactions, including the non-template control, were performed in triplicate. The presence of a single PCR product without any nonspecific amplicons was confirmed via agarose gel and melting curve analyses. The PCR product was sequenced by Sanger sequencing to confirm the amplification of 16S rRNA gene. All qPCR runs showed no PCR amplifications from non-template control and culture samples without adding 1H1Hc7 cells and had efficiency levels of. $\geq$ 98%, with an R^2 of >0.99.

Formate utilization of methanogen isolates

Six hydrogenotrophic methanogen strains previously isolated from sediments of the eastern Nankai Trough were cultured in a sulfate-free saline mineral medium (pH 7.2) supplemented with 20 mM sodium formate, 2 mM sodium acetate, 2 mM coenzyme M, 0.2 mM titanium(III) citrate, and 10 mM HEPES. Cultivation was performed at 25 °C under an N_2 atmosphere at atmospheric pressure. Methane in the headspace gas of triplicate cultures for each strain was quantified by gas chromatography as described above.

References

Cantalapiedra, C.P., Hernández-Plaza, A., Letunic, I., Bork, P., and Huerta-Cepas, J. (2021) eggNOG-mapper v2: Functional Annotation, Orthology Assignments, and Domain Prediction at the Metagenomic Scale. Mol Biol Evol 38: 5825-5829.

Chen, S., Zhou, Y., Chen, Y., and Gu, J. (2018) fastp: an ultra-fast all-in-one FASTQ preprocessor. Bioinformatics 34: i884-i890.

Katayama, T. & Kamagata, Y. (2015) Cultivation of Methanogens. In Hydrocarbon and Lipid Microbiology Protocols. New York NY: Springer, pp. 177–195.

Katayama, T., Nobu, M.K., Kusada, H., Meng, X.Y., Hosogi, N., Uematsu, K. et al. (2020) Isolation of a member of the candidate phylum 'Atribacteria' reveals a unique cell membrane structure. Nat Commun 11: 6381.

Lu, S., Wang, J., Chitsaz, F., Derbyshire, M.K., Geer, R.C., Gonzales, N.R. et al. (2020) CDD/SPARCLE: the conserved domain database in 2020. Nucleic Acids Res 48: D265-d268.

Seemann, T. (2014) Prokka: rapid prokaryotic genome annotation. Bioinformatics 30: 2068-2069.

Wick, R.R., Judd, L.M., Gorrie, C.L., and Holt, K.E. (2017a) Completing bacterial genome assemblies with multiplex MinION sequencing. Microbial Genom 3: e000132.

Wick, R.R., Judd, L.M., Gorrie, C.L., and Holt, K.E. (2017b) Unicycler: Resolving bacterial genome assemblies from short and long sequencing reads. PLoS Comput Biol 13: e1005595.
